# Tensile strength and impact strength of color modified acrylic resin reinforced with titanium dioxide nanoparticles

**DOI:** 10.4317/jced.53620

**Published:** 2017-05-01

**Authors:** Loghman Ghahremani, Saeed Shirkavand, Faezeh Akbari, Niloofar Sabzikari

**Affiliations:** 1 Assistant Professor, Department of Prosthodontics, Faculty of Dentistry, Urmia University of Medical Sciences, Urmia, Iran; 2 Graduate student, Department of Prosthodontics, Faculty of Dentistry, Urmia University of Medical Sciences, Urmia, Iran; 3 Dentistry student, Student Research Committee, Urmia University of Medical Sciences, Urmia, Iran

## Abstract

**Background:**

Poor mechanical properties are among the main limitations of acrylic resins. Addition of titanium dioxide (TiO2) nanoparticles to acrylic resin has been shown to improve its mechanical properties with an adverse effect on its color. Thus, this study sought to assess the tensile and impact strength of a color modified heat cure acrylic resin reinforced with TiO2 nanoparticles.

**Material and Methods:**

In this *in vitro*experimental study, 1wt% TiO2 nanoparticles were added to SR Triplex Hot heat-cure acrylic resin powder and mixed. Pigments and color fibers were also added and 18 samples were fabricated of this paste for tensile and impact strength testing (n=9) according to ISO5271. Eighteen control samples were also fabricated from the acrylic powder without any modification. Independent t-test was used for data analysis (*P*< 0.05).

**Results:**

The mean tensile strength of the reinforced group was found to be significantly higher (difference of 11 MPa) than that of the control group (*P*=0.001). The mean impact strength of the reinforced group was 7 MPa higher than that of the control group and this difference was statistically significant as well (*P*=0.001).

**Conclusions:**

The color modified acrylic resin reinforced with 1wt% TiO2 showed significantly higher tensile and impact strength compared to the conventional acrylic resin. Thus, TiO2 nanoparticles may be incorporated into color-modified acrylic resin powder to enhance its tensile and impact strength, given that they have no adverse effect on other properties.

** Key words:**Tensile strength, acrylic resins, titanium dioxide, impact strength.

## Introduction

Polymethyl methacrylate is among the most commonly used materials for the fabrication of removable complete or partial dentures. It is the main component of the denture base material and has easy handling and favorable esthetics. However, poor mechanical properties are among the main shortcomings of acrylic resins, making them susceptible to fracture. Attempts have been made to enhance the mechanical properties of acrylic resins by the incorporation of copolymers and cross-linking agents (1-6).

With the advances in nanotechnology, researchers attempted to reinforce the mechanical properties of acrylic resins by addition of metal oxide, carbon and glass fiber nanoparticles with promising results (7-11). Evidence shows that TiO2 nanoparticles can be added to dental materials to mimic the opaque color of natural teeth (12) or to decrease bacterial adhesion (13). However, despite excellent mechanical properties, it could not enhance the mechanical properties of composite resins (14-16). But, the situation seems to be different for the acrylic resins. Hernandez *et al.* showed that heat-cured Acralon, a polymethyl methacrylate resin, in combination with TiO2 had higher strength than the conventional acrylic resin for the fabrication of temporary crowns. However, they only measured the Knoop hardness number and did not compare the mechanical properties of the reinforced and control acrylic resins (17). In another study, Shirkavand and Moslehifard (1) demonstrated that addition of 1wt% TiO2 nanoparticles reinforced the acrylic resin but adversely affected its color. Thus, considering the extensive use of acrylic resins and their poor mechanical properties, this study sought to assess the effect of addition of 1wt% TiO2 on the tensile and impact strength of color-modified acrylic resin. The null hypothesis was that the addition of TiO2 nanoparticles would have no effect on the tensile and impact strength of color-modified acrylic resin compared to conventional acrylic resin.

## Material and Methods

This *in vitro*, experimental study was conducted on 36 samples in the two groups of test and control (n=18) and four subgroups of 9. Sample size was calculated to be 9 samples in each subgroup according to a study by Shirkavand and Moslehifard 1 and considering α=0.05 and 80% power of study.

-Fabrication of samples for the test group.

**Figure 1:**
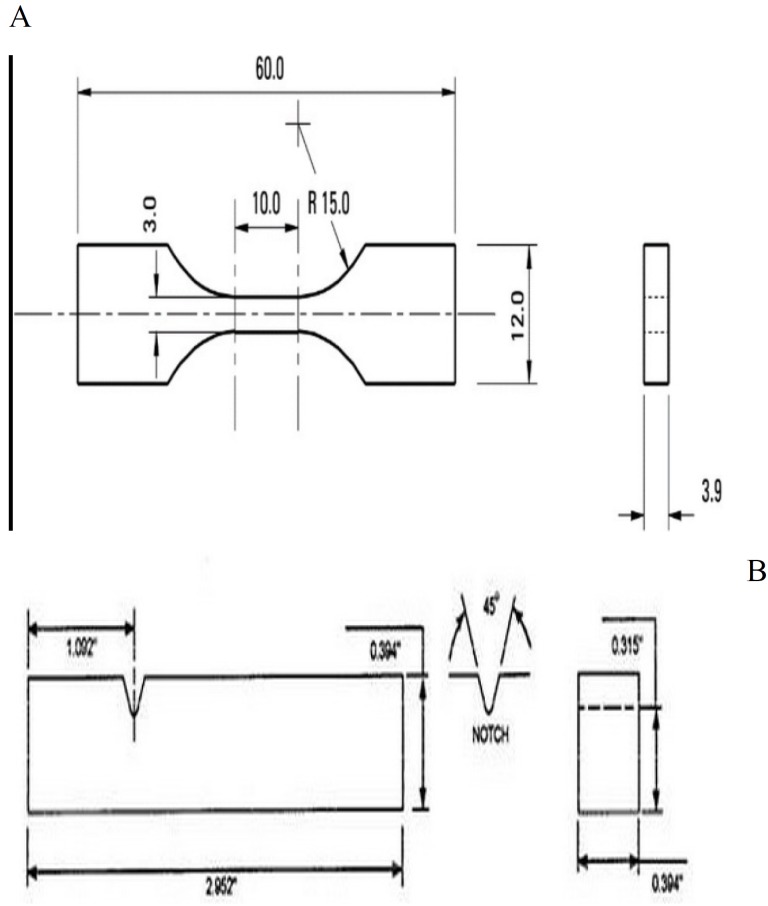
A) Shape and dimension of samples for the tensile strength testing B) Shape and dimension of samples for the impact strength testing.

The SR Triplex Hot heat-cure acrylic resin (Ivoclar Vivadent Inc. Schaan, Liechtenstein) powder was well mixed with 1wt% TiO2 nanoparticles (nanosav,Tehran,iran) in an ultrasonic mixer to obtain a homogenous blend. Color pigments and fibers were also added. The powder was mixed with the liquid as recommended by the manufacturer, applied to a mold and flasked. After heating, the samples were finished and polished using Emery paper (mehrdent,Tehran,iran) and were accurately measured by a digital caliper (Guanglu, Strikhlu, Germany) with 1mm accuracy and 25cm measuring capacity. For the measurement of tensile strength, dumbbell-shaped samples (n=9) were fabricated according to ISO 5271 (18) with the dimensions shown in figure 1A. For assessment of impact strength, cubic samples (n=9) were fabricated with dimensions shown in figure 1B with a notch.

-Fabrication of samples for the control group:

Eighteen control samples were also fabricated of SR Triplex Hot heat-cure acrylic resin (Ivoclar Vivadent Inc. Schaan, Liechtenstein) powder and liquid according to the manufacturer’s instructions with no modification in the powder. The process of fabrication was the same as that in the test group and samples were fabricated in two forms of dumbbell-shaped and cubic-shaped (n=9) with the same dimensions explained above for the measurement of tensile and impact strength, respectively.

**Figure 2:**
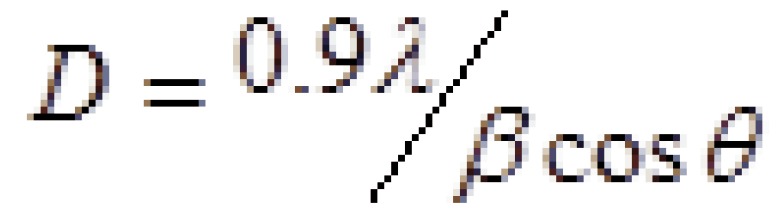
Equation.

**Figure 3:**
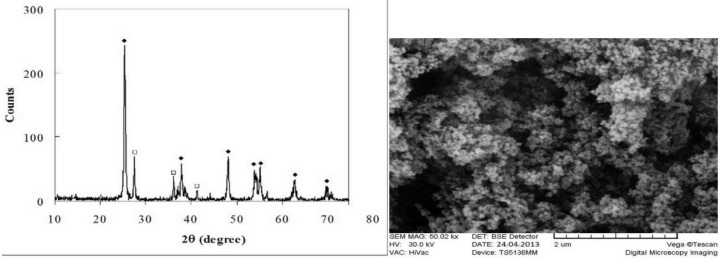
SEM micrograph and X ray diffraction analysis of TiO2 nano-powder used in this study.

-Assessment of morphology and structure of the modified acrylic powder:

To assess the morphology of the acrylic powder mixed with TiO2 nanoparticles, a scanning electron microscope (SEM; VE-GA\\TESCAN-LMU and VEGA\\TESCAN-XMU , Brno - Czech Republic) was used. For phase identification of TiO2 na-no-powder, X-ray diffraction was performed (X’pert MPD, Philips, Eindhoven, the Netherlands) with CuKa radiation at a wave-length of 1.542°A, 2θ=10-80° and step size of 0.03. The size of each nanoparticle was estimated using the Scherrer equation as follows, (Fig. 2):

Where β is the full width at half maximum of the diffraction peak in radiant, λ is the X ray wavelength in nm, L is the mean size of particles in nm and Θ is the peak diffraction angle in radiant. Based on this formula, the size of TiO2 nanoparticles was 20-30nm and the dominant phase was anatase (Fig. 3).

-Measurement of tensile strength:

Dumbbell-shaped samples were placed in a universal testing machine (Z100, Zwick Roell, Ulm, Germany) and fixed to the clasps. Tensile load was applied to the samples from both sides and increased until fracture. The maximum load at fracture displayed on the monitor was recorded.

-Measurement of impact strength.

The cubic samples were placed in their respective place in Izod universal testing machine (Z100, Zwick Roell, Ulm, Germany) and firmly fixed to the clasps in such a way that the notch was positioned between the clasps. Repeated strokes were applied to the samples with increasing load until fracture. The maximum load at fracture displayed on the monitor was recorded.

-Statistical analysis.

The mean and standard deviation (SD) of tensile and impact strength values were calculated in each group. SPSS version 20 was used for data analysis. Normal distribution was confirmed by Shapiro-Wilk test and independent t-test was used to compare the groups. (*P*<0.05 was considered statistically significant.)

## Results

Normal distribution of the data was tested by the Shapiro-Wilk test, which showed normal distribution of tensile strength (*P*=0.62) and impact strength (*P*=0.185) data. Thus, independent t-test was applied to assess the differences between the two groups.

**Figure 4:**
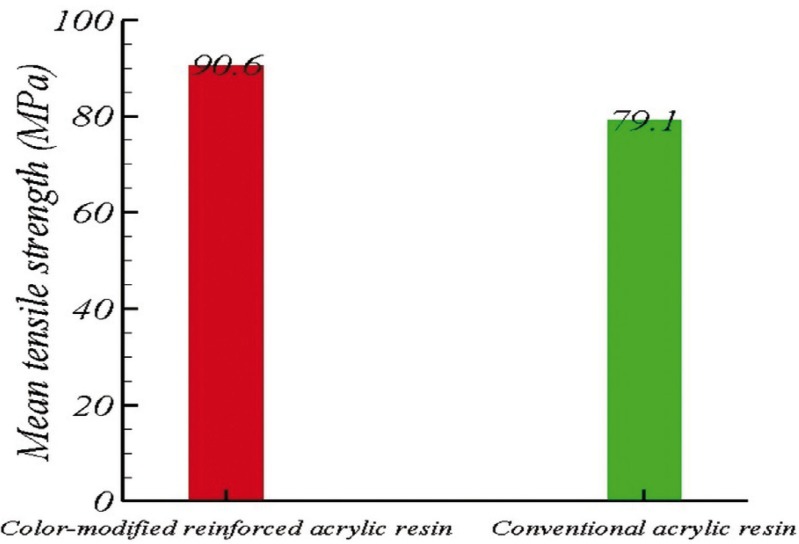
The mean tensile strength of the color-modified reinforced and conventional acrylic resin groups.

The mean tensile strength was 90.6±5 MPa in the color-modified reinforced group and 79.1±3 MPa in the conventional acrylic group (Fig. 4). The tensile strength of the modified group was found to be higher than that of the control group by 11.48 MPa. According to t-test, this difference was statistically significant (*P*=0.001).

**Figure 5:**
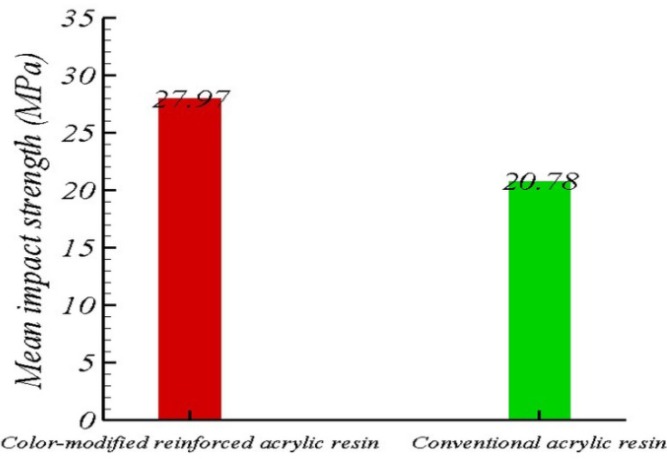
The mean impact strength of the color-modified reinforced and conventional acrylic resin groups.

The mean impact strength was 27.96±1.57 MPa in the color-modified reinforced group and 20.77±2 MPa in the conventional acrylic group (Fig. 5). The impact strength of the modified group was found to be higher than that of the control group by 7.52 MPa. According to t-test, this difference was statistically significant (*P*=0.001).

## Discussion

Acrylic resins are among the most commonly used dental materials. They are mainly used for the fabrication of acrylic denture bases with successful results attributed to their optimal esthetics and biocompatibility. However, their poor physical properties particularly their low tensile and impact strength are among their major drawbacks responsible for their easy fracture or cracking (2,3). Johnston *et al.* (4) stated that 68% of dentures break within their first year of clinical service. In the oral cavity, strong repetitive masticatory forces cause fatigue and eventual fracture or cracking of dentures. Extra-orally, dentures may break, chip or crack if dropped (5,6). Evidence shows that maxillary dentures often break at the midline due to fatigue or impact (5,7); while 80% of mandibular dentures break due to impact (5).

Incorporation of nanoparticles into the acrylic resin powder was recently suggested to improve its physical properties. Shirkavand and Moslehifard (1) showed that addition of 1wt% TiO2 nanoparticles reinforced acrylic resin but changed its color. Thus, the current study was conducted to assess the effect of addition of TiO2 to color-modified acrylic resin powder on its tensile and impact strength in comparison with conventional acrylic resin. The results showed that the mean tensile and impact strength values of the modified acrylic resin were significantly higher than those of the control group. Thus, addition of 1wt% TiO2 particles to acrylic resin significantly increased its tensile and impact strength and the null hypothesis of the study was refuted. This result is probably attributed to the fact that following the incorporation of nanoparticles into acrylic powder, the applied load is mainly tolerated by these particles. The polymer matrix provides structural integrity and load distribution. Thus, crack propagation is inhibited.

Acosta-Torres *et al.* (19) stated that metal oxides are suitable for addition to acrylic polymers to enhance their mechanical properties and showed that titanium dioxide nanoparticles improved the mechanical properties of resins (19), which was in agreement with our findings.

In the study by Shirkavand and Moslehifard (1), TiO2 nanoparticles in 0.5, 1 and 2wt% concentrations were added to polymethyl methacrylate acrylic resin. They showed that addition of 1wt% nanoparticles had the greatest efficacy for enhancement of tensile and impact strength. Thus, we added 1wt% TiO2 nanoparticles to acrylic powder. As seen on the SEM micrograph (Fig. 3), addition of 1wt% nanoparticles yielded a homogenous blend, which further explains the optimal results obtained. Our results were similar to those of Shirkavand and Moslehifard (1); however, in their study, addition of TiO2 adversely affected the color while we fixed this problem by addition of pigments. Sodagar *et al.* (20) also showed that addition of TiO2 nanoparticles improved the flexural strength of acrylic resin, which was in line with our findings.

Small sample size was a limitation of this study. Therefore, further studies with larger sample sizes are required to better elucidate this topic. Also, the effect of addition of TiO2 on other properties of acrylic resin can be an interesting topic for future studies.

## Conclusions

The color modified acrylic resin reinforced with 1wt% TiO2 showed significantly higher tensile and impact strength compared to the conventional acrylic resin. Thus, TiO2 nanoparticles may be incorporated into color-modified acrylic resin powder to enhance its tensile and impact strength, given that they have no adverse effect on other properties.
